# Adult Small Intestinal Intussusception Due to Bleeding Lipoma: A Rare Case Report (with Video)

**DOI:** 10.3390/reports8040221

**Published:** 2025-10-31

**Authors:** Mariafelicia Valeriani, Ciro De Martino, Marianna Capuano, Agostino Fernicola, Francesco Cerfolio, Giovanni Aprea, Giuseppe Palomba

**Affiliations:** 1 Azienda Ospedaliera Universitaria “San Giovanni di Dio e Ruggi d’Aragona”, 84131 Salerno, Italy; 2 Department of Clinical Medicine and Surgery, “Federico II” University, Via Pansini 5, 80131 Naples, Italy

**Keywords:** intussusception, gastrointestinal bleeding, intestinal obstruction, emergency surgery

## Abstract

**Background and Clinical Significance**: Adult small intestinal intussusception is rare and, in this population, is usually secondary to a pathological lead point, such as benign or malignant masses. The symptoms are non-specific, and patients frequently present with intermittent abdominal pain, diarrhea, nausea, vomiting, and, in rare cases, bleeding. There are currently no specific guidelines. Surgery remains the gold standard treatment. **Case Presentation**: We report the case of a 55-year-old female affected by Shone’s syndrome, presenting to the emergency department with melena, severe anemia, diffuse abdominal pain, weakness, and palpitations. Diagnostic tests showed active bleeding in the small intestine and a subocclusive condition. Urgent surgery was performed, revealing an intussusception. **Conclusions**: A multidisciplinary approach is essential for its management. Surgical resection is the only option in these cases, as it allows histological examination to rule out malignancy.

## 1. Introduction and Clinical Significance

Adult small intestinal intussusception is rare, representing 5% of all intussusceptions and causes 1–5% of intestinal obstructions in this population [1,2]. This condition is more common in the pediatric population, and in adults, it is usually secondary to a pathological lead point [1,2]. In the small intestine, the causes are various and may be due to intra- or extraluminal masses such as polyps, lipoma, or other intestinal lesions (approximately 30–55% of cases are malignant), gallstones through a cholecystectomy fistula, which can cause intestinal obstruction, jejunal diverticula, foreign bodies, phytobezoars, postsurgical adhesions, endometriosis, and inflammatory bowel disease [2,3].

The symptoms of this disease are non-specific, and patients frequently present with intermittent abdominal pain, diarrhea, nausea, and vomiting. In rare cases, these symptoms may also be associated with intestinal bleeding [2,4]. Abdominal computed tomography (CT) is necessary for a diagnosis, showing “sausage-shaped,” “target,” or “cuppy” signs with a layering effect [5,6,7]. There are no guidelines on the optimal treatment of this pathology; given the uncertainty of the lead point, surgical resection of the involved tract is the treatment of choice [1,4].

We report a 55-year-old female patient with a rare case of intussusception due to an acute bleeding small intestinal lipoma with occlusive symptoms. This report aims to underline the exceptional rarity of the case presented, since, to our knowledge, only a few cases of simultaneous onset of these two complications have been reported in the literature. This study was in accordance with the SCARE criteria [8].

## 2. Case Presentation

A 55-year-old woman presented to the emergency department with melena, diffuse abdominal pain, weakness, and palpitations. Her medical history was notable for Shone’s syndrome, atrial fibrillation (AF) with documented episodes of ventricular tachycardia, for which she was on treatment with Coumadin, chronic heart failure, hypothyroidism, gonarthritis, and hearing loss. Initial laboratory assessments revealed severe anemia (hemoglobin, 6.8 g/dL), coagulopathy (international normalized ratio [INR], 3.66; prothrombin activity, 20%; activated partial thromboplastin time [aPTT], 43.2 s), and an elevated lactate level (2.6 mmol/L).

Blood transfusions and warfarin antagonists (vitamin K) were administered to achieve hemodynamic stability. Subsequent esophagogastroduodenoscopy (EGD) revealed no evidence of active, recent, or prior bleeding.

EGD and contrast-enhanced computed tomography (CT) of the abdomen and pelvis were performed as part of the diagnostic workup once upper gastrointestinal bleeding had been excluded.

**Figure 1:**
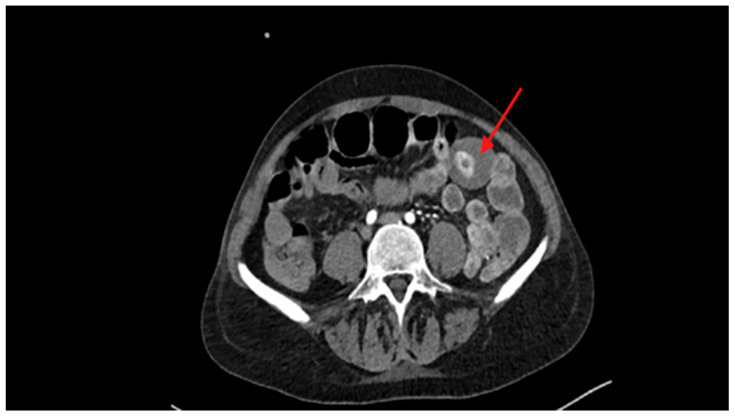
CT scan identified an intensely hyperdense endoluminal spot in the small intestine (red arrow).

A CT scan identified, during the arterial phase, an intensely hyperdense endoluminal spot in the small intestine within the meso-hypogastric region, highly suggestive of a bleeding polyp or lipoma [Figure 1]. In the subsequent portal and delayed phases, the lesion showed a further increase in density and size, with progressive opacification of the most caudal intestinal loops, consistent with distal passage of the contrast medium due to active luminal bleeding.

**Figure 2:**
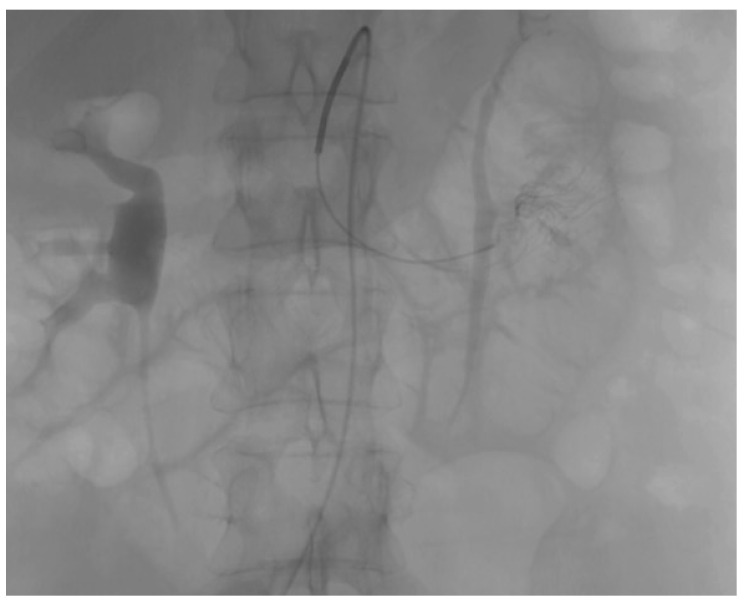
Radiological angiography.

Subsequently, angiography was performed, which revealed active contrast extravasation from the terminal branches of the ileocolic artery, prompting immediate superselective radiological angioembolization (Figure 2).

Despite embolization, 24 h later, persistent anemia (hemoglobin, 6.8 g/dL) and the onset of an acute obstructive abdominal condition necessitated urgent surgical intervention.

**Figure 3:**
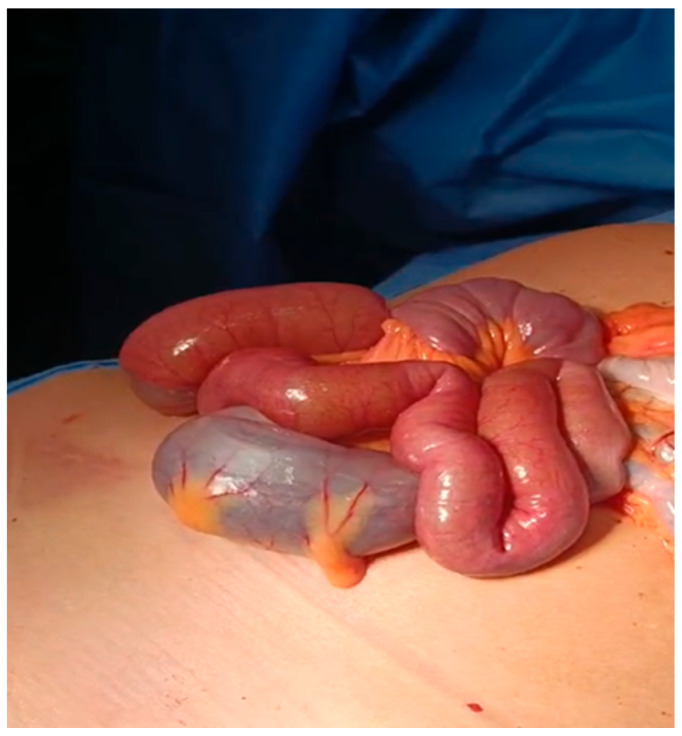
Median laparotomy, which revealed immediately the presence of intestinal dilation with the co-existence of an ischemic tract.

**Figure 4:**
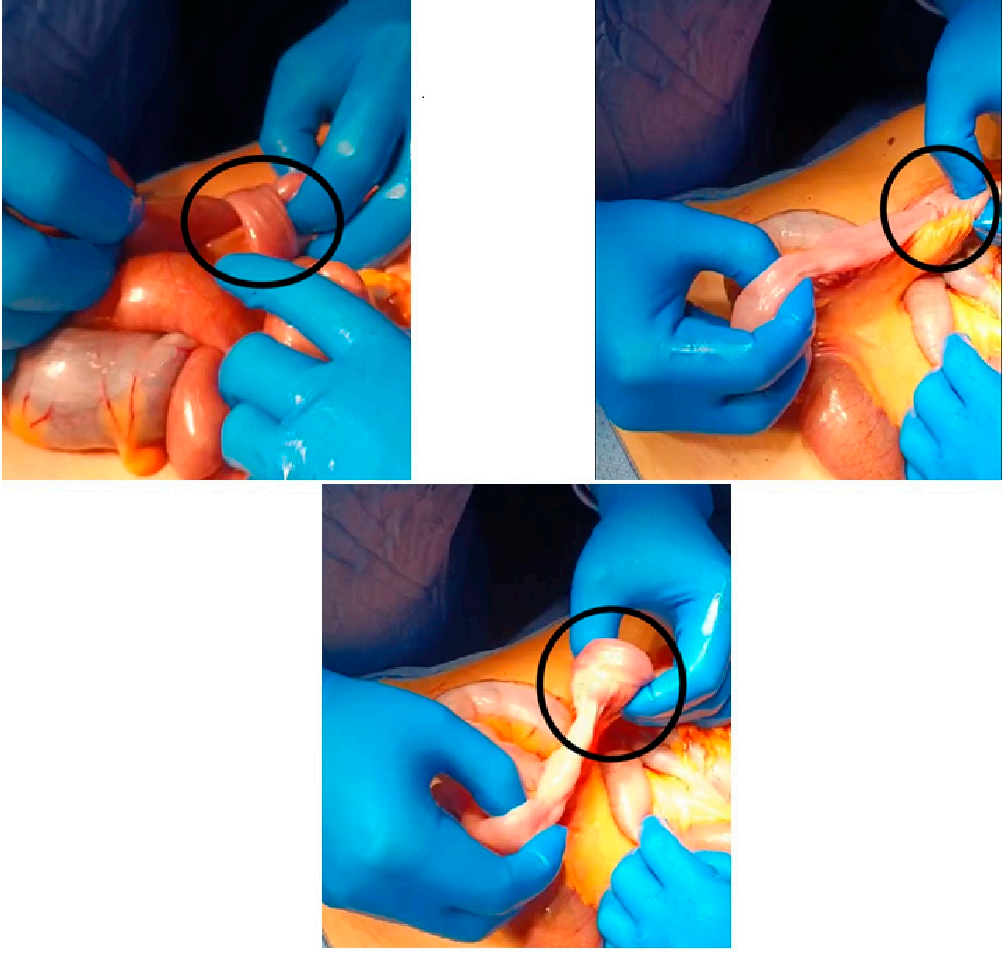
These figures show the stages of intussusception reduction. The black circles show the circumferential invagination of the intestine due to the lead point.

A median laparotomy was elected as the surgical approach, which revealed immediately the presence of intestinal dilation with the co-existence of an ischemic tract (Video S1) (Figure 3). The assessment of intestinal ischemia was performed intraoperatively by evaluating both the serosa color and superior mesenteric artery pulsation. Furthermore, it showed intussusception of the small intestine approximately 50 cm from the Ligament of Treitz (Figure 4).

A 2-cm intraluminal neoformation, previously described on CT, was identified within the affected segment. The involved segment (approximately 50 cm) was resected due to the undetermined origin of the lesion, and a lateral–lateral anisoperistaltic jejunal-jejunal anastomosis using a mechanical stapler, ensuring proper alignment and perfusion of the bowel segments, was performed.

On postoperative day 1 (POD 1), the patient experienced highly responsive atrial fibrillation and recurrence of anemia, both of which were managed with medical therapy and transfusions, respectively. Anticoagulant therapy with enoxaparin sodium and the patient’s original anticoagulant regimen were gradually reintroduced according to the established protocol after cardiology consultations. Parenteral nutrition was maintained until POD 3, followed by the initiation of a liquid diet on POD 4, with gradual advancement to a soft diet. The patient was discharged on POD 6 with improvement in clinical and laboratory parameters, including a stable increase in hemoglobin levels (hemoglobin, 10.1 g/dL). At the two-week outpatient follow-up, the patient reported complete resolution of symptoms.

Histological examination revealed an extensively ulcerated polypoid mass of 2.5 cm × 1.5 cm × 1 cm with fibrin-necrotic-granulocytic exudate and chronically active granulation tissue with reactive vascular proliferation. It was characterized by submucosal proliferation, histologically composed of mature adipose tissue (S100+). Histological examination of the resected specimen confirmed the diagnosis of intestinal submucosal lipoma.

Table 1 shows the timeline from emergency department admission to postoperative outpatient follow-up.

## 3. Discussion

Intussusception is more common in children [1]. In adults, it is rare, accounting for approximately 5% of all intussusception cases and 1–5% of intestinal obstruction cases, and represents a challenge in terms of diagnosis and management in this population [9,10].

In children, the causes are mainly idiopathic, while adult intussusception is usually secondary to a pathological lead point, such as benign or malignant masses, gallstones through a cholecystectomy fistula, jejunal diverticula, foreign bodies, phytobezoars, postsurgical adhesions, endometriosis, and inflammatory bowel disease [2,3,11]. In 30–55% of cases, this condition is caused by malignant tumors [10]. These data correlate with the prevalence of this event between the fifth and sixth decades of life [11]. In our case, an intestinal submucosal lipoma caused both bleeding and intestinal obstruction with intussusception. Intestinal lipomas are rare benign mesenchymal tumors representing approximately 2–5% of all gastrointestinal tumors [12,13]. In 20–30% of cases, they are present in the small intestine, in 60–70% of cases, in the colon and ileum–colon, and in a small percentage of cases, in other locations [3,12,13,14,15,16,17].

The term intussusception refers to the circumferential invagination of a segment of the gastrointestinal tract into the lumen of an adjacent segment. This may involve the proximal segment invaginating into the adjacent distal segment, or vice versa [9,18]. A lead point that alters or blocks normal peristalsis, causing a focal area of abnormal motility, triggers this process [9,16]. If not treated promptly, as intussusception progresses, vascular damage may occur, resulting in ischemia, necrosis, and potential perforation [7,9,18].

In children, there is often a triad of symptoms with colicky pain, palpable mass, and “currant jelly” stools [19]. The symptoms of adult intussusception are non-specific, and patients frequently present with intermittent abdominal pain, diarrhea, nausea, and vomiting [9,20]. Rarely, these symptoms may also be associated with intestinal bleeding, and some patients present with weight loss or palpable abdominal masses [2,4,15]. Adult intussusception due to intestinal lipoma is a rare condition [21,22,23,24]. In our case, the patient presented with simultaneous significant acute gastrointestinal bleeding and obstructive symptoms. Owing to the non-specificity of the symptoms, diagnosis often appears to be a difficult challenge. Indeed, our patient is bleeding, and anemia played a confounding role in the initial diagnosis.

Computed tomography (CT) is the gold standard for diagnosing adult intussusception. CT shows a detailed view of the characteristic “target,” “cuppy,” or “sausage-shaped” mass, identifying lead points and signs suggestive of malignancy, such as irregular borders or invasion into adjacent structures [5,7,25].

Endoscopy may be used in cases involving the colon or distal small intestine; however, it is not sufficient for definitive diagnosis and treatment.

The management of intussusception in adults is complex because its etiology is malignant in most cases [25,26]. There are currently no specific guidelines. Surgery remains the gold standard treatment.

Surgical resection involves en-bloc removal of the affected intestinal tract with appropriate margins, followed by primary anastomosis when possible [18,20,21,22,23,24,25,26,27].

The choice between an open or laparoscopic approach depends on the surgeon’s expertise, patient stability, and suspected pathology [18,28]. In our choices, we involved a multidisciplinary team composed of an interventional radiologist, anesthetist, hemostasiologist, and emergency surgeon. Given the uncertain location of the bleeding, we first performed embolization to stabilize the patient. Subsequently, we performed a surgical resection with anastomosis.

The prognosis of intussusception in adults is determined by both the etiology of the lead point and the timeliness of treatment.

Delayed diagnosis may cause intestinal ischemia, necrosis, perforation, and peritonitis, and malignant etiology may increase morbidity and mortality. Our shared choices with the multidisciplinary team allowed us to manage this complex and critical case effectively.

This case presents a rare instance of intussusception and acute gastrointestinal bleeding in an adult caused by a submucosal lipoma of the small intestine. Specific guidelines should be developed to improve prognosis, quality, and standardization of care.

## 4. Conclusions

Submucosal intestinal lipomas can cause bleeding, obstruction, and intussusception. In our case, this condition presented with multiple complications simultaneously. The multidisciplinary approach was effective in managing this complex disease. Surgical resection is the only possible option in these cases, as it allows histological examination to exclude malignancy.

## Figures and Tables

**Table 1:** The timeline from emergency department admission to postoperative outpatient follow-up.

Date/Day	Clinical Event	Details
Day 0—Emergency department—H15:00	Presentation admission	55-year-old female. Symptoms: melena, diffuse abdominal pain, weakness, and palpitations. Medical history: Shone’s syndrome, atrial fibrillation, chronic heart failure and hypothyroidism.
Day 0—H15:14	Laboratory tests	Severe anemia (Hb 6.8 g/dL), INR 3.66, lactate 2.6 mmol/L. Blood transfusions administered.
Day 0—H18:00	Upper endoscopy	No evidence of active or recent bleeding.
Day 0—H20:45	CT scan	Hyperdense intraluminal lesion in the small intestine, suspicious of bleeding lipoma.
Day 0—H23:30	Angiography	Active contrast extravasation from ileocolic branches → selective embolization performed.
Day +1—H00:00	Clinical evolution	Persistent anemia and onset of acute obstructive abdominal condition.
Day +1—H4:00	Urgent surgery	Midline laparotomy revealed ischemic tract and intussusception ~50 cm from Treitz. En-bloc Resection of ~50 cm segment with lateral–lateral jejunal-jejunal anastomosis.
POD 1	Early complications	Recurrence of atrial fibrillation and anemia, managed with medication and transfusions.
POD 3–4	Recovery of nutrition	Transition from parenteral nutrition to liquids and then soft diet.
POD 6	Discharge	Clinical and laboratory improvement with stable hemoglobin levels.
2-week follow-up	Outpatient evaluation	Patient asymptomatic, hemoglobin stable and complete symptom resolution.
Histology	Final diagnosis	Submucosal intestinal lipoma.

## Data Availability

The data presented in this study are available from the corresponding author upon request.

## Supplementary Materials

The following supporting information can be downloaded at https://www.mdpi.com/article/10.3390/reports8040221/s1, Video S1: intraoperative video.
